# Eco-friendly bupropion detection sensor with co-formulated dextromethorphan in AUVELITY tablet and spiked plasma

**DOI:** 10.1038/s41598-024-80227-2

**Published:** 2024-11-26

**Authors:** Shrouk E. Algmaal, Amr M. Mahmoud, Shereen A. Boltia, Yasser S. El-Saharty, Nermine S. Ghoniem

**Affiliations:** Analytical Chemistry Department, Faculty of Pharmacy, Cairo University, Kasr El-Ainy St., Cairo, ET-11562 Egypt

**Keywords:** Biochemistry, Biological techniques

## Abstract

Molecularly Imprinted Polymers (MIPs) are synthetic materials designed to selectively recognize and bind to specific target molecules. The process of determining Bupropion (BUP) using MIPs involves preparing the MIP, extracting the target molecule, and conducting subsequent analysis. A bio-inspired MIP-based electrochemical sensor was developed to detect BUP, utilizing the specific binding of MIPs to Bupropion molecules, enabling precise and sensitive detection. The combination of molecular imprinting and electrochemistry in this approach allows for the development of a highly reliable and effective sensor specifically designed for BUP detection. In this method, copolymerization conditions were carefully optimized to ensure selectivity and sensitivity in detecting BUP. Different monomers, including o-phenylenediamine, 4-aminophenol, L-dopa, and 1,4-phenylenediamine, were explored, with the best interaction observed for L-dopa and 1,4-phenylenediamine. Consequently, their copolymer was implemented to create selective MIPs through a straightforward electropolymerization process on a disposable pencil graphite electrode (PGE) substrate for BUP detection. The functionality of the copolymer of L-dopa and 1,4-phenylenediamine as an electroactive copolymer in preparing electro-polymerized MIP films was investigated for the first time. This was demonstrated by constructing a novel electrochemical sensor for the selective recognition of BUP in different matrices. The interactions between L-dopa and 1,4-phenylenediamine, used as functional monomers, and the template were studied experimentally using UV spectroscopy. BUP was used as the template, and the copolymer was electrografted onto PGE. The constructed sensor was characterized using cyclic voltammetry (CV), and BUP binding to the MIP cavities was measured indirectly with differential pulse voltammetry (DPV) using a ferrocyanide/ferricyanide redox probe. A linear and repeatable response was displayed by the sensor across a range of 1.0 × 10⁻^13^ M to 1.0 × 10⁻^11^ M of BUP, with a limit of detection of 3.18 × 10⁻^14^ M. The sensor demonstrated robust selectivity for BUP over interfering drugs, such as dextromethorphan, in pharmaceutical dosage forms and spiked human plasma. The environmental impact of the proposed approach was evaluated using green analytical chemistry principles, including the Green Analytical Procedure Index (GAPI) and the Analytical GREEnness (AGREE) metric.

## Introduction

**Fig. 1:**
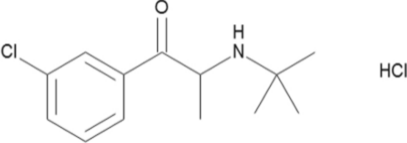
The chemical structure of bupropion.

Bupropion, also known as ( ±)-2-(tert-butylamino)-3´-chloropropiophenone hydrochloride, is an aminoketone derivative (Fig. 1). It is a second-generation antidepressant that differs from traditional tricyclic antidepressants in its neurochemical action. BUP has been shown to be a selective inhibitor of catecholamine (dopamine and noradrenalin) neuronal reuptake, with no effect on indolamine (serotonin) reuptake and no inhibitory action on monoamine oxidase 3. One of the main uses of bupropion is the treatment of severe depressive illness. It is an aminoketone that falls under the category of an inhibitor of norepinephrine-dopamine reuptake. This drug can cause toxicity to the central nervous system and cardiovascular system in overdose cases, which can cause dysthymias and seizures. Due to their widespread use in people with underlying depression, antidepressants are often linked to overdoses. Nevertheless, because bupropion shares structural similarities with amphetamines and synthetic cathinones, purposeful misuse, including insufflation, has also been documented^1^ 0.16,926 Bupropion overdoses were reported to the American Association of Poison Control Centers in 2020. Merely around 50% of these exposures (8,668) consisted of a single exposure, resulting in eight fatalities^2^ so it is important to develop easy and accurate method to determine Bupropion .Constipation, headache, dry mouth, nausea, sleeplessness, and dizziness are the most frequent side effects^3^. The development of a novel detection method for Bupropion is crucial due to the growing demand for precise, reliable, and rapid analytical techniques in pharmaceutical analysis, therapeutic drug monitoring, and forensic toxicology. Existing methods for Bupropion detection often encounter challenges such as complex sample preparation, lengthy procedures, and susceptibility to matrix interferences, which can affect the accuracy and sensitivity of results. This novel method overcomes these issues by offering enhanced selectivity, sensitivity, and reduced analysis time, advancing the field of analytical chemistry.

The Molecularly Imprinted Polymer (MIP)-based sensor developed in this research provides unique advantages over existing technologies. It is specifically tailored to the molecular structure of Bupropion, offering superior selectivity by creating specific recognition sites that mimic the target interactions, thereby minimizing interference from similar compounds. Unlike traditional methods such as chromatography, which require extensive sample preparation, the MIP-based sensor offers a rapid and straightforward detection process, making it ideal for real-time monitoring and point-of-care applications. The MIP sensor exhibits high sensitivity due to its strong binding affinity for Bupropion, enabling the detection of low concentration levels often missed by other techniques. Its cost-effective fabrication and reusability without significant performance loss make it an economically sustainable alternative to disposable sensors. Additionally, the sensor’s robustness against common matrix interferences in complex samples ensures consistent and reliable results.

Overall, this MIP-based sensor addresses current gaps in Bupropion detection methods and sets a new standard in sensor technology. It offers a practical, efficient, and highly selective tool for drug analysis with significant implications for pharmaceutical quality control, clinical diagnostics, and environmental monitoring, marking a substantial advancement in the field.

Methods for determining BUP in biological and pharmaceutical samples have primarily been established using chromatography and electrochemistry. For chromatographic methods, liquid chromatography coupled with tandem mass spectrometry^4^, and high-performance liquid chromatography with a spectrofluorometric detection^2^ are used. Just a few examples of electrochemical sensors that allow for the determination of BUP in the context of electroanalytical methods have been reported thus far. These examples include ion-selective PVC membrane electrodes^5^ , bulk glassy carbon electrodes^6^, and a carbon screen-printed electrode that has been modified with graphene oxide, gold nanoparticles, molecularly imprinted polymer as an electrochemical impedance spectroscopy sensor^7^ and plasma-enhanced chemical vapor deposition (PECVD) process on a screen-printed electrode SPE ^8^. Bupropion is often combined with various drugs, particularly antidepressants. The newly FDA-approved AUVELITY tablet is a combination of Bupropion and Dextromethorphan designed for the treatment of major depressive disorder. Only two HPLC methods have been reported for determining this combination^9,10^ , and no electrochemical methods have been documented. Consequently, there is a significant need for an environmentally friendly, quick, simple, and selective process that can detect BUP, either alone or in combination with other antidepressants. This procedure should not include complex equipment, extensive extraction times or sample collecting procedures, or costly solvents .Currently, the creation of affinity sensors based on Molecularly Imprinted Polymers (MIPs) is gaining significant attention^11–14^. Molecular imprinting technology is a synthetic polymer-based approach used to construct three-dimensional recognition sites for specific chemicals. These recognition sites are designed to match the size, shape, and chemical activity of the target molecule, making them highly specific and tailored to the intended analyte. MIPs are increasingly used to mimic natural receptors, making them valuable for applications such as drug screening and in vivo monitoring^14–17^. Recognizing elements that interact with particular target analytes are the heart of electrochemical sensors. Making specific binding sites in synthetic polymers with molecular templates is a well-proven and economical method known as molecular imprinting^12^, and was effective in creating sensitive sensor systems for chemical, biological, environmental, and therapeutic detections.^12,18,19^. The usual method for creating MIPs is to polymerize functional monomers with template molecules present. The target analyte’s functional groups and shape are complemented by the 3D-cavities created by template removal. MIPs act similarly to natural biological receptors like enzymes and antibodies because of their selectivity towards analyte molecules and their recognition methods. But MIPs are appealing in the realm of chemical sensing because of their high chemical and thermal stability, economical cost, and simplicity of synthesis. Electrochemical sensors generate qualitative or quantitative signals by utilizing data from electrochemical reactions between an electrode and an analyte. Conductometric sensors measure changes in the electrical conductivity of a solution caused by the interaction between the analyte and the sensor surface, making them commonly used for detecting ionic species. Amperometric or voltammetric sensors detect current changes resulting from redox reactions at the electrode surface when a specific potential is applied. These sensors are known for their high sensitivity and are widely used to detect various analytes, including gases and biomolecules. Potentiometric sensors measure the potential difference between two electrodes without drawing current, allowing for the detection of specific ions in a solution. They are frequently employed in pH meters and ion-selective electrodes^20^.

The polymerization process involves the chemical reaction of small molecules called monomers to form larger, chain-like structures known as polymers. This process can occur through various techniques, each tailored to achieve specific properties and applications of the resulting polymer. Common polymerization methods include in situ polymerization, which occurs directly within the desired matrix; interfacial polymerization, where reactions occur at the interface of two immiscible phases; matrix polymerization, which involves embedding the polymer within a solid matrix; emulsion polymerization, which produces polymers in a dispersed phase within an emulsion; and suspension polymerization, which uses suspended droplets to form bead-like polymers. Each technique offers unique advantages in controlling polymer properties, such as structure, size, and functionality, making polymerization a versatile tool in material science and sensor development. Electropolymerization offers several advantages over traditional polymerization techniques. A significant benefit is that it occurs at room temperature, eliminating the need for light, oxidants, or free radical initiators to initiate the process. This method produces polymers that form adherent, homogeneous, and conformal films on the electrode surface, with easily and precisely controllable properties, including thickness. The technique’s simplicity, minimal sample requirements, rapid analysis, and portability make electrochemical sensors highly appealing for various applications^21^.The process of electropolymerization can employ a variety of conducting polymers. including polypyrrole, carbazole, polyphenanthroline, azobenzene, poly(1,10-phenanthroline-5,6-dione), poly(9,10- phenantherenequinone), and poly(pyrrole-2-carboxylic acid)^22^. 1,4-Phenylenediamine (1,4-PDA) is a commonly used monomer in the synthesis of molecularly imprinted polymers (MIPs)^23,24^. Its popularity is due to the amino groups (-NH₂) in its structure, which can form strong interactions with the functional groups of the template molecule during polymerization^25,26^. These interactions create highly specific binding sites within the MIP, making it ideal for capturing the target molecule. The chemical structure of 1,4-PDA facilitates the formation of multiple hydrogen bonds and other interactions with various templates. Moreover, derivatives of phenylenediamine are widely utilized in pharmaceutical and analytical industries due to their versatile binding capabilities^25,27^.

Dopamine and L-dopa are ideal building blocks for creating electro-polymerizable polymers due to their ability to form strong, non-covalent bonds with other substances, such as through stacking, electrostatic interactions, and hydrogen bonding^28^. L-dopa, a naturally occurring amino acid essential for the production of neurotransmitters like dopamine, is particularly suitable for developing MIPs aimed at detecting neurotransmitters or delivering drugs for neurological disorders.

One significant challenge in utilizing electrochemical (bio) sensors is the presence of interfering substances, such as cells and proteins, in physiological fluids like urine, plasma, and saliva. These substances can non-specifically adhere to the sensor’s surface, leading to inaccurate readings. Additionally, the low concentration of target chemicals in these fluids complicates detection, resulting in poor specificity, low signal-to-noise ratios, and potential false-positive results. As highlighted in several recent reviews^29–34^, “fouling” remains a primary obstacle to the practical application of electrochemical (bio) sensors.

To mitigate fouling, researchers are exploring non-fouling chemistries. By modifying the sensor’s surface with highly hydrated and polar materials, the adherence of unwanted substances can be significantly reduced^33^. Catecholamine polymers, derived from dopamine and L-dopa, have gained popularity in chemical, biological, and material sciences. These polymers exhibit excellent adhesion, durability, antifouling properties, and biocompatibility, making them ideal for surface modification and material engineering. Inspired by mussel adhesion, these polymers are highly hydrophilic and contain functional groups such as amine, imine, and phenolic hydroxyl groups^35–37^.

Poly(levodopa) is particularly promising for creating antifouling surfaces due to its ability to resist nonspecific protein adsorption in its zwitterionic state. This study presents the development of a new electrochemical sensor designed for rapid measurement of BUP levels in plasma samples. The sensor was fabricated by electropolymerizing a copolymer of L-dopa and 1,4-phenylenediamine onto a disposable pencil graphite electrode (PGE).

Pencil graphite electrodes have gained extensive use across various industries due to their inherent electrochemical properties and cost-effectiveness. In the field of electroanalysis, PGEs have been employed to identify both organic and inorganic compounds from a wide range of samples, from simple matrices like water to complex biological fluids such as human urine or serum. A recent review highlighted the application of PGEs in the analysis of environmental samples, detailing the historical use of graphite pencils as electrodes. The electrode material of PGEs, derived from standard writing pencils, offers numerous advantages, including low cost, easy availability, and safety for both users and the environment, as they are nontoxic^38–40^. After optimizing factors such as pH, contact time, and the number of electropolymerization cycles, the sensor exhibited excellent sensitivity, selectivity, and a low detection limit for BUP, even in the presence of its co-formulated drug, dextromethorphan, and in spiked human plasma samples.

## Material and methods

### Instruments

Electrochemical measurements were performed using NOVA 1.11 software on an Autolab potentiostat/galvanostat equipment (model: PGSTAT204, Metrohm, Utrecht, Netherlands), a platinum counter electrode, and an Ag/AgCl reference electrode. The spectrophotometric measurements were performed using the Shimadzu EPMA-1610, Tokyo, Japan UV–visible double beam spectrophotometer.

### Reagents and materials

Pure BUP (certified to contain 99%) according to USP official method^41^ was kindly obtained by Merck Company (Cairo, Egypt). 105 mg BUP and 45 mg dextromethorphan per tablet were the indicated amounts for the commercially marketed AUVELITY extended-release tablets (BN: 81,968–045-30) made by Axsome Therapeutics, Ind., New York. L-dopa, 1,4-phenylenediamine, potassium ferricyanide (K_3_[Fe(CN)_6_]), potassium ferrocyanide (K_4_[Fe(CN)_6_]), glacial acetic acid and potassium chloride were acquired from Sigma-Aldrich (Darmstadt, Germany). The remaining substances and reagents were of analytical grade. The water purification system, called New Human Power I (Human Corporation, Seoul, South Korea), produced double-distilled water. The necessary pH range of 5.0 to 8.0 was achieved by freshly prepared phosphate buffer. The holding company for biological products and vaccines (VACSERA, Cairo, Egypt) provided the blank human plasma.

### Investigation of the monomer-BUP interaction

**Fig. 2:**
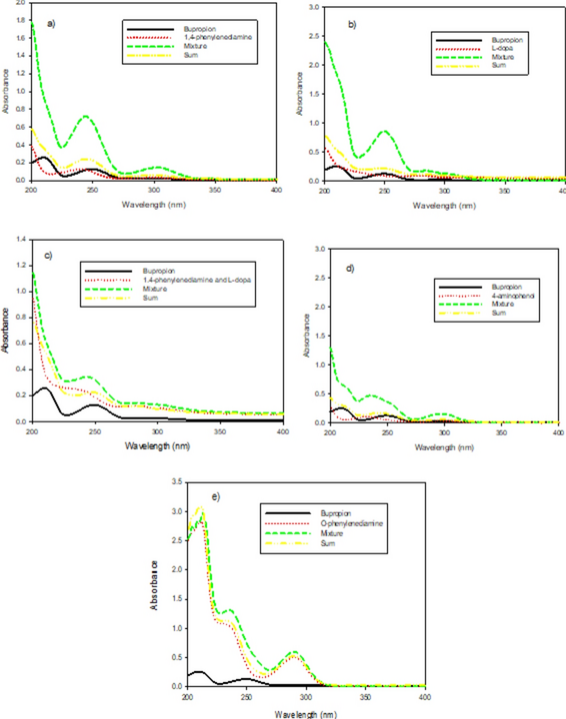
The UV spectra of 1.0 × 10^–5^ M bupropion and the investigated monomers: (**a**) 1,4 phenylenediamine, (**b**) L-dopa, (**c**) equimolar mixture of L-dopa and 1,4-phenylenediamine, (**d**) 4-aminophenol & (**e**)* o*-phenylenediamine, in phosphate buffer pH 7.0 along with their pre-polymerization complexes prepared at 1:1 molar ratio and the calculated sum.

Various monomers, including o-phenylenediamine, 4-aminophenol, L-dopa, and 1,4-phenylenediamine, were tested as shown in Fig. 2a–e, but the best interaction was observed with L-dopa and 1,4-phenylenediamine as shown in Fig. 2a–c. Therefore, their copolymer was utilized in this study. Using a UV spectrophotometer, the interaction between BUP and the L-dopa and 1,4-phenylenediamine copolymer was analyzed. The spectra of 1.0 × 10^–5^ M of each compound, both individually and in an equimolar mixture with the monomer template in phosphate buffer (pH 7), were recorded. The spectra were collected over a wavelength range of 200–400 nm.

### Electropolymerization of copolymer at the PGE surface

First, the bare PGEs were cleaned of any contaminants by washing them in a solution of methanol and water. They were then exposed to a nitrogen stream to dry out. Next, the PGEs were attached to serve as working electrodes, and two centimetres of the electrode were immersed in a solution that contained L-dopa (10.0 mM), BUP (10.0 mM), 1,4 phenylenediamine (10.0 mM), and phosphate buffer (pH 7). After that, oxygen was extracted from the solution by purging it with a nitrogen stream for 15 min. Ten voltammetric cycles were applied across an Ag/AgCl electrode from -0.1 V to 0.9 V at a scan rate of 50 mV/s in order to perform in-situ electropolymerization. Next, for 20 min under gentle shaking in the glacial acetic acid/methanol (2:8, v/v) washing solution, the polymerized electrodes, dubbed as MIP/PGE, were left in the solution. In order to utilize them in the next stage, the electropolymerized electrodes were dried and rinsed with water. Cyclic voltammetry was used for electrochemical characterization in an equimolar solution of 10 mM [Fe (CN)6] ^3-/4-^ with 0.1 M KCl.

### Electrochemical measurements of BUP

Prior to conducting the analysis, the MIP/PGE was submerged in the drug solution ranging from 1.0 × 10^−13^ M to 1.0 × 10^−11^ M for 10 min while being stirred, and it was then rinsed for 30 s with water. A solution containing 5.0 mM [Fe(CN)_6_]^3−/4−^ in phosphate buffer (pH 5.0) was measured using differential pulse voltammetry at room temperature over a potential window of − 0.1 to 0.8 V relative the Ag/AgCl reference electrode. The normalized reduction in the current peak height of the redox probe was plotted against the corresponding BUP concentration, ranging from 1.0 × 10^−13^ M to 1.0 × 10^−11^ M, to construct the calibration curve.

### Application of a pharmaceutical dosage form

After weighing ten tablets, each containing 105 mg of BUP and 45 mg of dextromethorphan, the tablets were ground into a fine powder. An amount of this powder equivalent to 27.6 mg of BUP was dissolved in 100 mL of phosphate buffer (pH 5.0). This solution was then diluted using phosphate buffer (pH 5.0) and filtered to achieve final concentrations of 1.0 × 10^−11^ M, 1.0 × 10^−12^ M, and 1.0 × 10^−13^ M of bupropion.

The electrochemical measurements were subsequently performed as previously described, and the BUP concentration was estimated using the regression equation.

### Application to spiked human plasma

One millilitre of a 1 × 10^−3^ M BUP and 0.43 × 10^−3^ of its co-formulated drug dextromethorohan standard solutions were added to 1 mL of human plasma, and the mixture was ultrasonically mixed for twenty minutes. The samples were then purified by precipitating the plasma proteins with the addition of 3.5 mL of acetonitrile. Ultimately, the samples underwent a 30-min, high-speed centrifugation. A phosphate buffer solution (pH 5.0) was used to dilute several aliquots from the supernatant to 10 mL, resulting in final concentrations for BUP of 5.0 × 10^−12^ M, 7.0 × 10^−12^ M, and 8.0 × 10^−12^ M. The average recoveries were then computed following the electrochemical measurements.

### Method validation

The recommended approach was verified respect to linearity, accuracy, precision, and limits of quantification and detection, in compliance with the standards of the International Council for Harmonization of Technical Requirements for Pharmaceuticals for Human Use (ICH)^42^. Under ideal circumstances, linearity was assessed by measuring six BUP concentrations. The linear correlation was obtained by plotting the normalized drop in current peak height against concentration within the range of 1.0 × 10^−13^ M to 1.0 × 10^−11^ M. The analytical method’s accuracy is the measure of the closeness of agreement between the value which is accepted either true value or an accepted reference value and the value found was expressed as the % recovery of the drug at different levels in standard solutions. The evaluation of precision was conducted by quantifying three different drug concentrations within the linearity range, for both repeatability (on the same day) and intermediate precision (on three different days). Next, the relative standard deviation percentage (% RSD) was used to express the results, and then the limits of detection (LOD) and quantification (LOQ) were also computed.

### Interference

The selectivity of the sensor and its capacity to quantify BUP in the presence of medicines that interfere with it were studied using co-administered pharmaceuticals, namely dextromethorphan and diazepam. The measurement was made of the current peak height shift in the coexistence of interfering ions (1.0 × 10^−4^ M), which was one million times the concentration of BUP under ideal circumstances.

## Results and discussion

### UV spectrophotometry assessing the interactions between the template and monomer

Spectrophotometric methods are typically quick, economical, and effective means of screening various complexes created by optimizing the stoichiometric ratio between a template and a monomer^43–45^. Hence, spectrophotometric analysis at pH 7 was used to investigate monomers and potential complexes in phosphate buffer solution. The mixtures of BUP/1,4-phenylenediamine and BUP/L-dopa produced an absorption spectrum, as seen in Fig. 2a–c, which showed the formation of a complex between BUP and L-dopa and 1,4-phenylenediamine; these were then compared to the computed sum of the spectrums BUP/L-dopa and BUP/1,4-phenylenediamine^44–46^. On the other hand, when BUP was combined with o-phenylenediamine and 4-aminophenol, no discernible shifting was seen.

### PGE/MIP surface characterisation

**Fig.3:**
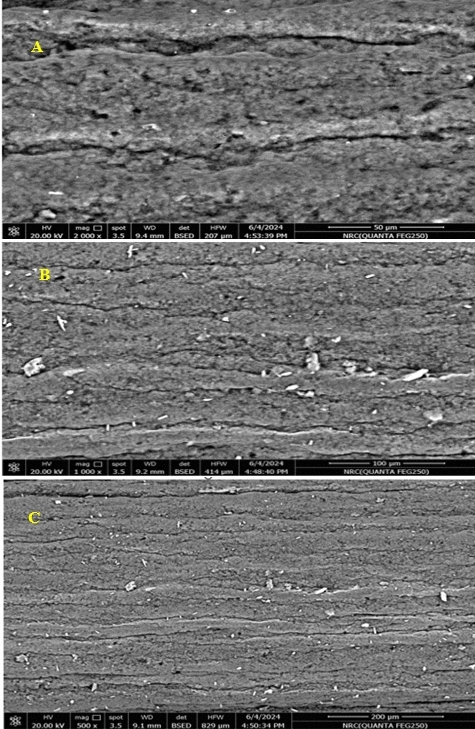
(**a**–**c**): PGE/MIP surface characterisation by SEM.

The data are displayed in Fig. 3(a–c), where the photos obviously reveal the presence of an evenly dispersed polymer layer over the PGE surface, signifying the effective electropolymerization of MIP. Scanning electron microscopy (SEM) was used to examine the surface morphology.

#### Characterization of the PGE/MIP using electropolymerization process

**Fig. 4:**
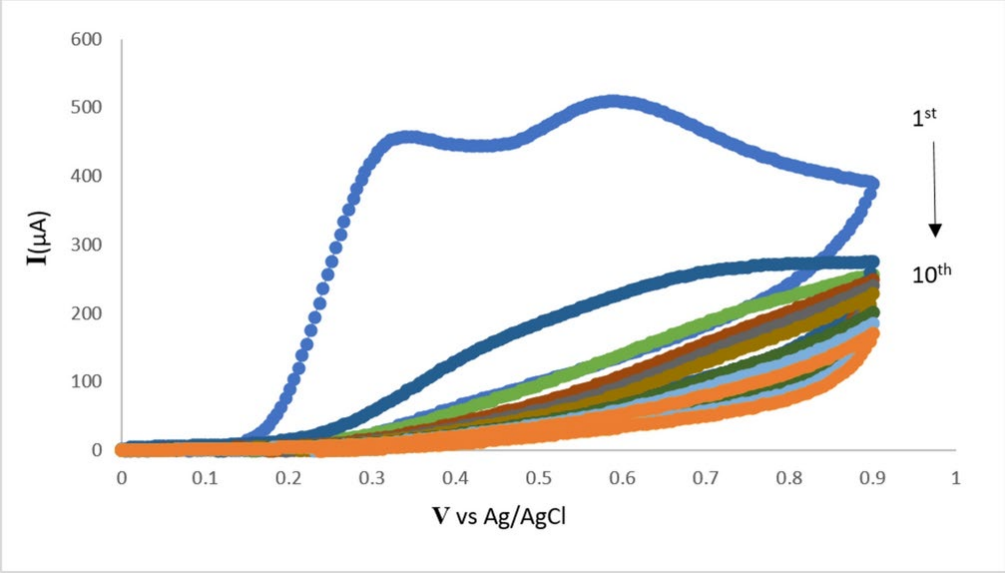
The cyclic voltammogram of the L-dopa and 1,4 phenylenediamine electropolymerization process over 10 cycles.

Numerous investigations were conducted to thoroughly examine the electro-polymerization of dopamine and its analogues. The resulting polymeric films were found to be highly effective MIPs for the intended analytes^37,47^. Nevertheless, the electro-polymerization of L-dopa and 1,4-phenylenediamine and their use as a functional monomer in the creation of MIP electrochemical sensors have not yet been covered in any of these studies. Using the CV methodology, a novel process for the electro-polymerization of L-dopa and 1,4-phenylenediamine was devised and meticulously refined^47^. PGEs were compared to other conventional carbon electrodes in terms of ease of use, affordability, and environmental friendliness. They were also evaluated for their practicality and disposable qualities^39^. The recorded voltammogram of the copolymer electro-polymerization after 10 cycles of employing an applied voltage of -0.1 V to 0.9 V at a scan rate of 50 mV/s is displayed in Fig. 4. First, it was suggested that oxidation, intramolecular cyclization, and subsequent polymerization take place. This was demonstrated by the anodic peak current’s rapid drop after the first cycle, as shown in Fig. 4. Following that, the peak current steadily dropped as the number of voltammetric cycles increased until it decreased after ten cycles, indicating that the PGE surface had completely insulated and that an adherent polymeric film had formed^48^. In order to produce BUP/PGEs, electro-polymerization was carried out with BUP acting as the template molecule.

**Fig.5:**
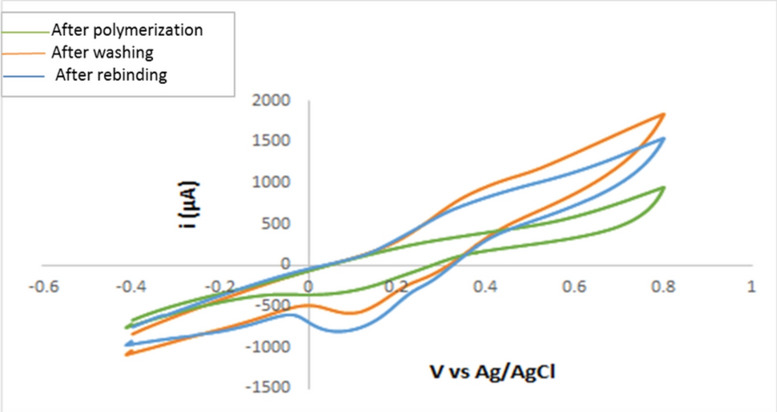
The cyclic voltammograms of the electrode of L-dopa and 1,4 phenylenediamine for a redox probe solution (equimolar 5 mM [Fe(CN)6]^3−/4−^ in 0.1 M KCl) after polymerization, after washing, and after rebinding.

Numerous researchers have embraced the CV technique^2,49,50^, to examine multiple stages of polymerization through the use of a redox probe for ferrocyanide/ferricyanide, as demonstrated in Fig. 5, The electropolymerization step has clearly resulted in a decline in the peak current value at the BUP/PGE surface prior to template removal. This illustrates how a film of copolymer insulates the entire PGE surface, hindering electron transfer^2^. The redox probe peaks persisted after the PGE washed with an extraction solvent composed of glacial acetic acid and methanol (2:8, v/v). The observation might potentially be clarified by removing the template medication, which created pores in the MIP matrix that permitted the redox probe to pass through. These pores were blocked when the BUP molecules were rebound, resulting in a notable reduction in the redox probe signal. These outcomes also demonstrated the capacity to create an imprinted cavity that the elution process could use to adsorb the analyte. Electrochemical Impedance Spectroscopy (EIS) was employed to monitor changes on the electrode surface during the modification process. The charge transfer resistance (Rct) of the PGE/MIP sensor was initially high (653.5 Ω) after polymerization and before elution, indicating the formation of a dense polymer layer. Upon template removal, the Rct significantly dropped to 77 Ω, suggesting the formation of binding cavities. After incubation in the BUP solution, the Rct increased to 226 Ω, confirming the successful rebinding of BUP to the MIP cavities.

**Fig.6:**
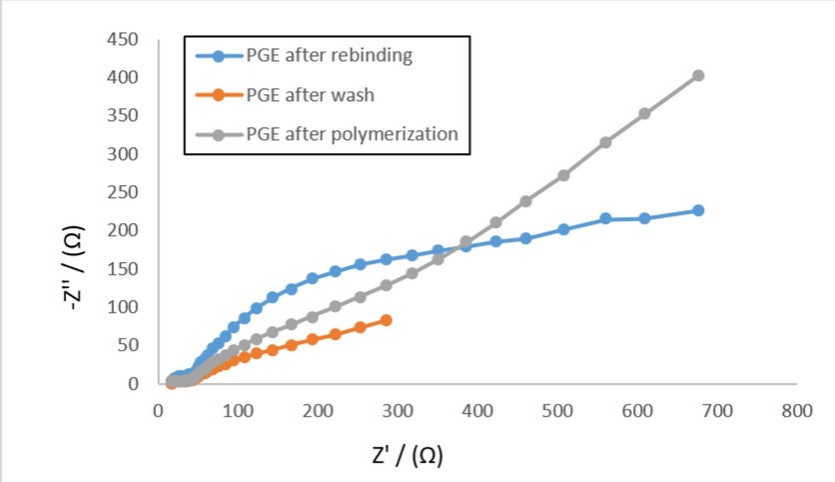
: The Nyquist plots of the proposed electrode during the modification process (after polymerization, after washing, and after rebinding) in the redox probe solution of equimolar 5.0 mM [Fe(CN)6]3 − /4 − in the presence of 0.1 M KCl at a frequency range of 100 kHz to 100 mHz.

These observations demonstrate that the polymerization process created an ultrathin polymer layer with high Rct, which decreased following the elution of the template, confirming cavity formation. The subsequent increase in Rct upon rebinding indicates the sensor’s effective recognition of BUP. The EIS results were in excellent agreement with the findings from the cyclic voltammetry and faradaic impedance tests, as shown in Fig. 6.

### Method optimization of electropolymerization

**Fig. 7:**
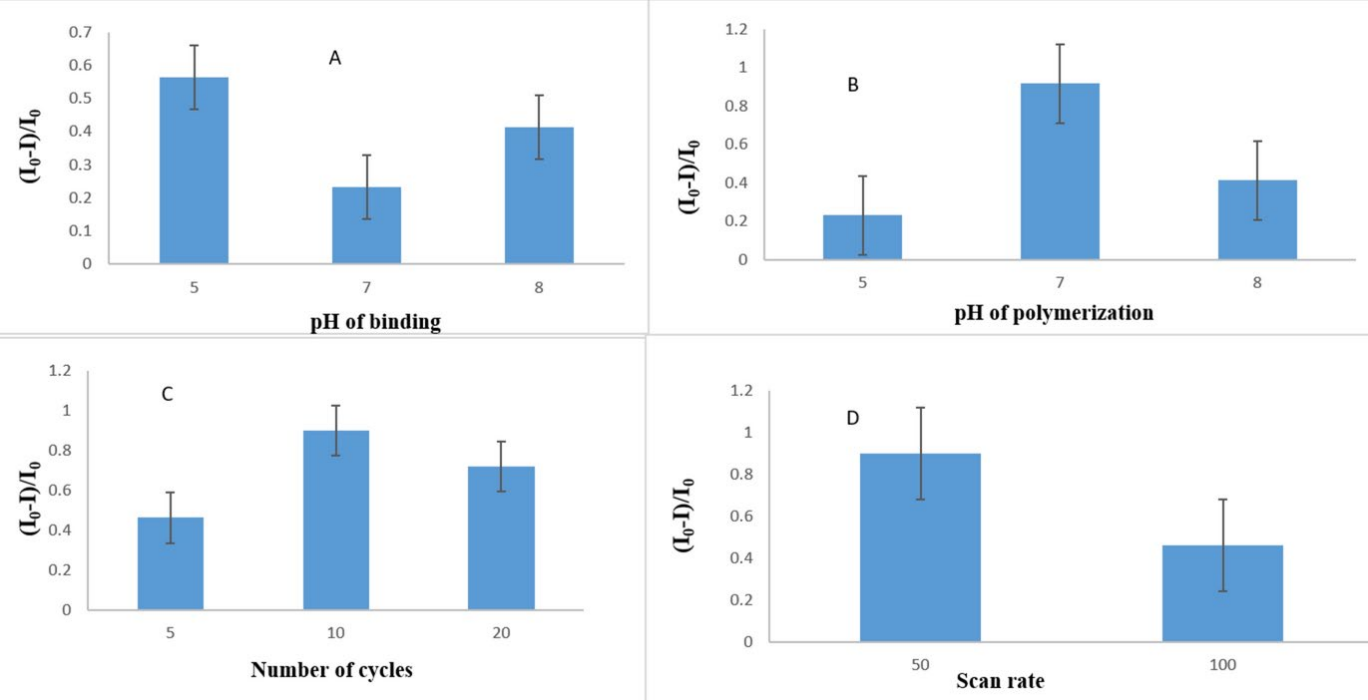
The optimization of various parameters affecting the electropolymerization. Process. (**A**) binding pH, (**B**) electropolymerization pH, and (**C**) number of cycles, (**D**) scan rate.

By enhancing the experimental circumstances, the electropolymerization procedure was refined to increase the sensitivity of the sensor^51–53^.One variable at a time, a univariate optimization research was used to assess intentional modest changes in the experimental parameters, such as the binding pH, the polymerization pH, and number of cycles. Results for each parameter examined at various levels are shown in Fig. 7. The binding pH had a significant impact on how well the drug bound to the polymer during binding the ideal reaction was shown to occur at pH 5 according to (Fig. 7A). Additionally, the pH played a significant influence in the behaviour of the polymerization process, the ideal reaction was found to occur at pH 7 according to (Fig. 7B), The maximal reaction was attained after 10 cycles, after which it decreased with more cycles (Fig. 7C), varied scan rate was used to change the current response, and 50 mV s − 1 was shown to be the greatest response (Fig. 7D).

As such, the optimization studies made it possible for the sensor response to the BUP to be maximized.

#### The analytical performance of the proposed BUP sensor

**Fig.8:**
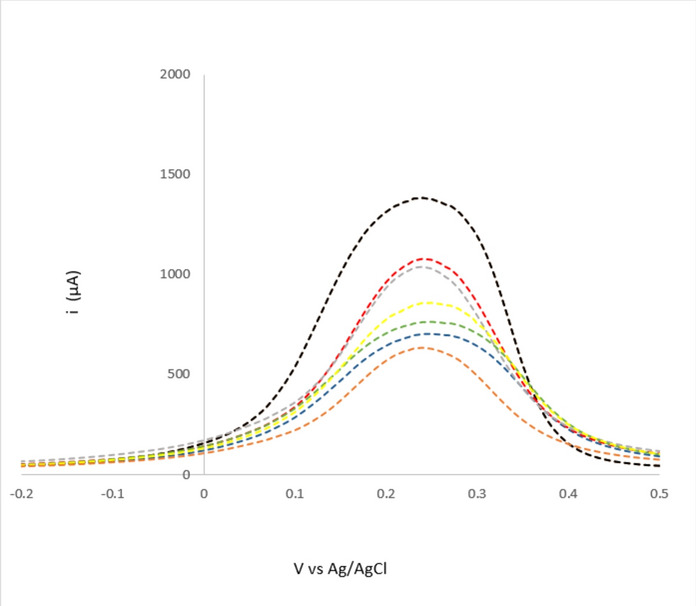
Differential pulse voltammograms of redox probe (equimolar of 5 mM [Fe(CN)6]^3−/4−^ in 0.1 M KCl) at MIP/PGE surface in the presence of various concentrations of BUP ranging from 1.0 × 10^− 13^ M to 1.0 × 10^−11^ M.

**Fig.9:**
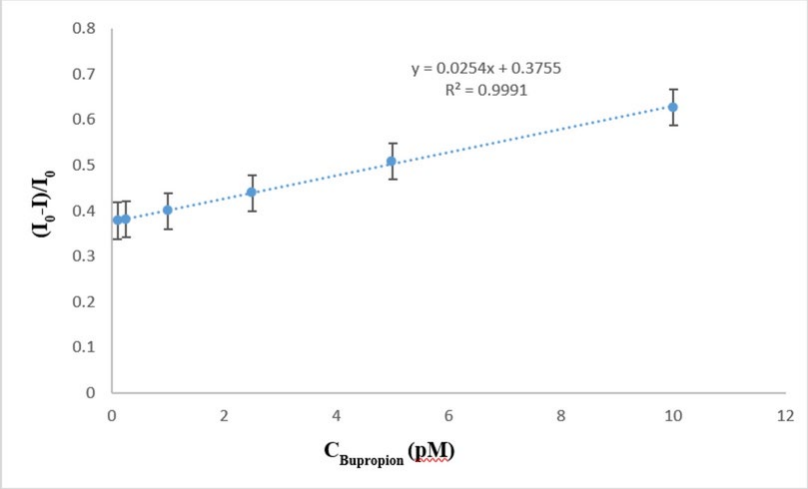
The calibration curve of BUP solutions ranging from 1.0 × 10^−13^ M to 1.0 × 10^−11^ M at optimum conditions.

Differential pulse voltammetry (DPV) was chosen over cyclic voltammetry (CV) for quantitative estimation of BUP using BUP/PGEs. DPV offered a superior current sensitivity and signal-to-noise ratio. The peak current obtained during the DPV measurement was used to quantify BUP concentration as shown in Fig. 8. The charging current before and after the application of pulse was used to calculate the current. Under ideal electrochemical circumstances, the procedure was discovered to be linear between 1.0 × 10^−13^ and 1.0 × 10^−11^ M. The method’s correlation coefficient was 0.9995. Table 1 and Fig. 9 provide a summary of the linearity results.

**Table 1:** The proposed electrochemical performance of the sensor based on IUPAC recommendations.

Parameter	Bupropion
Slope	0.0254
Correlation coefficient (r)	0.9995
Intercept	0.3755
Range of linearity (M)	1.0 × 10^−13^ M to 1.0 × 10^−11^ M
Accuracy (mean ± SD)	100.96±2.46
Precision (% RSD)	
Intraday precision	0.87
Interday precision	1.85
LOD	3.18 × 10⁻^14^ M.
LOQ	9.65 × 10⁻^14^ M.

Moreover, the accuracy of the suggested approach was shown by the analysis of three different BUP levels and their recovery%. The percentage RSD values were found to be within the allowed range for both intraday and interday precision, which must not exceed 2.5%^54^. This demonstrated that the suggested method was precise. Table 1 displays the findings for the LOD, LOQ, accuracy, and percentage RSD.

### Selectivity

**Fig.10:**
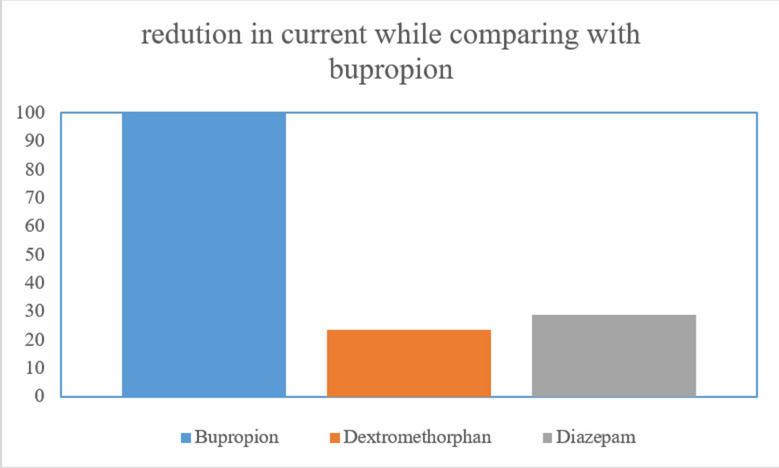
Selectivity bar graph to show reduction in current while comparing with bupropion.

Table 2 summarizes the outcomes of selectivity. The MIP selectivity was examined using dextromethorphan and diazepam, with a measured concentration of 1.0 × 10^−4^ M. The redox probe’s normalized current peak reductions for dextromethorphan and diazepam were found to be 23.39% and 28.94%, respectively. As a result, it was determined that these concurrently delivered medications had no effect on BUP’s DPV detection, demonstrating the MIP sensor’s great selectivity as shown in Fig. 10.

**Table 2:** Evaluation of the selectivity of the proposed sensor for BUP in the presence of interfering molecules.

Interferant	Concentration	Current reduction (%)
Dextromethorphan	1 × 10^–4^ M	23.39
Diazepam	1 × 10^–4^ M	28.94

### Application in dosage form and spiked plasma samples in the presence of its co-formulated drug dextromethorphan

The suggested MIP/PGE electrode was created to quantify BUP using AUVELITY a pharmaceutical tablet formulation. DPV measurements were conducted in triplicate, Table 3 displays the appropriate average recovery percentage with % RSD not exceeding 2.0. These results demonstrated that the method had an excellent recovery rate and demonstrated the great affinity of the synthesized MIP for BUP recognition.

**Table 3:** Quantitative estimation of BUP in tablets and plasma samples using the proposed MIP sensor in the presence of its co-formulated drug dextromethorphan.

Sample	Concentration of BUP (M)	Recovery% ± RSD%*
AUVELITY tablets	1.0 × 10^–11^	99.03 ± 0.04
	1.0 × 10^–12^	96.10 ± 0.19
	1.0 × 10^–13^	99.24 ± 0.94
	Mean ± SD 98.12 ± 1.43	
Spiked human plasma	5.0 × 10^–12^	104.12 ± 0.33
	7.0 × 10^–12^	104.16 ± 0.06
	8.0 × 10^–12^	98.92 ± 0.51
	Mean ± SD 102.40 ± 2.46	

*Values are the average of three independent analysis of the same sample (n=3).

Human plasma samples were tampered with varying known amounts of BUP standard solution and its co-formulated drug dextromethorphan to thoroughly test the MIP/PGE sensor’s suitability for practical analysis. The suggested sensor was used to measure BUP, and the average percentage recovery ranged from 98.92 to 104.16% as shown in Table 3. These findings reveal that the suggested PGE/MIP sensor holds significant promise for the quick and accurate measurement of BUP in actual plasma samples without interference from the biological matrix or its co-formulated drug. The suggested electrochemical MIP sensor’s applicability for determining the targeted drug in actual pharmaceutical formulation samples was ensured by statistical analyses that compared the outcomes of the proposed and official method using the F-test and the Student’s t-test. The computed Student’s t-test and F-test values were less than their relative tabulated ones, and the P value was adjusted at 0.05. This suggested that there was no statistically significant difference between the suggested and official methods, and that the suggested sensor was reliable for quantifying BUP as shown in Table 4. A comparison between the suggested sensor and other sensors for BUP determination is shown in Table 5. From this comparison, it can be inferred that the proposed electrochemical method is more sensitive and convenient. It offers cost efficiency, simplicity in sensor preparation, and ease of measurement, making it superior in most aspects compared to previous publications. This advantage likely stems from the application of indirect detection of BUP binding to MIP cavities using differential pulse voltammetry with a ferrocyanide/ferricyanide redox probe.

**Table 4:** Statistical comparison between the proposed method and official method for the BUP determination in AUVELITY dosage form.

Parameters	AUVELITY tablet dosage form	
	Proposed method	Official method ^a^^28^
Mean ± SD	98.12 ± 1.43	99.84 ± 1.46
n	9	3
Variance	2.04	2.13
Student’s T-test (2.228)^b^	1.77	–
F value (4.46)^b^	1.04	–

^a^HPLC method utilizing C18 column and mobile phase composing of methanol: tetrahydrofuran: phosphate buffer (pH 7) (39:11:50, by volume) at flow rate of 1.1 mL /min and UV detection at 250 nm^28^.

^b^The number in parentheses are the respective tabulated values for t and f at (p = 0.05).

**Table 5:** Comparison of some analytical parameters of the BUP quantitative determination using proposed sensor and some other sensors.

Technique	Electrode	Linearity range (M)	Real sample	LOD (M)	Reference
MIP (This study)	MIP/PGE	1.0 X 10^−13^ to 1.0 X 10^−11^	Tablets/plasma	5.4 × 10^−14^	This study
Potentiometric	MWCNT/CPE	5.0 X 10^–6^ to 1.0 X 10^–3^	Tablets	3.5 X 10^–6^	^4^
Adsorptive stripping voltammetry	Glassy carbon electrode	1.0 X 10^–6^ to 5.6 X 10^–6^	-	1.27 X 10^–7^	^2^
Electrochemical impedance spectroscopy (EIS)	MIP/AuNPs/GO/SPCE	2 X 10^–9^ to 990 X 10^–9^	Urine	0.5 × 10^–9^	^5^
Plasma-enhanced chemical vapor deposition (PECVD)	screen-printed carbon electrode modified with plasma polymerized acrylonitrile nanofilms	0.63 X 10^–6^ to 10 X 10^–6^	river water, groundwater, and sewage, as well as synthetic urine and blood serum	0.21 X10^–6^	^6^

### Greenness evaluation

**Fig.11:**
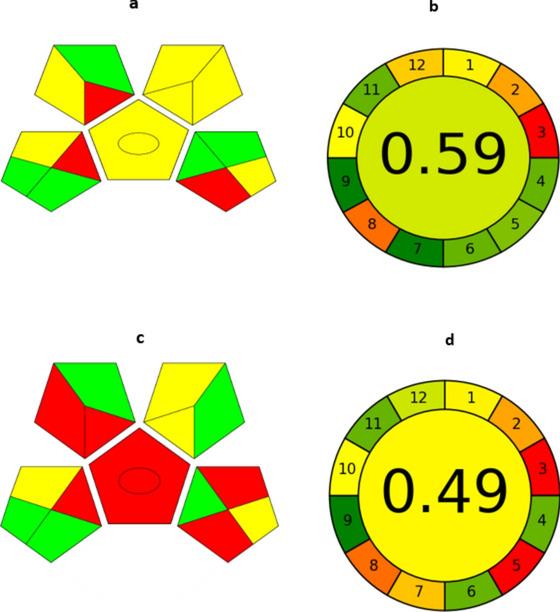
GAPI and AGREE pictograms for assessment of greenness profile assessment for the proposed method and reported method^5^ respectively.

In order to evaluate the provided approaches’ adherence to green analytical chemistry for the purpose of determining the substance under study, the greenness profile of each method was evaluated. For that purpose, the green analytical procedure index (GAPI) and the analytical greenness measure (AGREE) were employed. A new metric called GAPI was created to evaluate how each stage of the developed analytical process affects the environment. The environmental consequences of each step in the analytical procedures from sample preparation and collection to the final step of sample quantification and analysis is depicted as five pentagrams using three distinct color codes, red, yellow, and green, which stand for low, medium, and high environmental impact^55^.The introduced procedures had five green due to no sample preservation , no sample transport, no additional treatment, energy ≤ 0.1 kWh per sample and Hermetic sealing of analytical process, seven yellow due to storage under normal conditions, simple procedure for sample preparation, green solvents used in extraction (methanol), amount of reagents or solvents 10–100 mL, methanol health NFPA = 2, methanol flammability NFPA = 3, waste 1–10 mL and only three red zones due to off-line sample collection, macro extraction, no treatment of waste, (Fig. 11a) meanwhile the reported method^5^ had five green, four yellow, and six red zones owing to the health hazardous, non-green solvents are used, emission of vapour to the atmosphere, off-line sample collection, macro extraction and no treatment of waste in the reported method (Fig. 11c). The AGREE tool, which provides an overall score to represent the twelve GAC qualities, is thought to be a comprehensive and user-friendly assessment tool. The final score is shown in the centre of a colored pictogram that has 12 pieces, created from the resulting scale from 0 to 1. Each area of the pictogram is colored using a color scale ranging from green to red based on the developed method environmental impact. The overall score, which is represented by a green tint and values near to 1, indicates that the evaluated technique is more environmentally friendly^56^. The AGREE pictogram for our suggested technique has a score of 0.59 (Fig. 11b), compared to 0.49 (Fig. 11d) for the reported method. The AGREE score of our presented technique was affected by factors such as the sample size, waste amount, and sample extraction from biological samples, as well as the flammability and toxicity of the solvents used. However, our method demonstrated higher GAPI and AGREE scores due to the reduced volume of wasted solvents, the use of safe reagents, lower electrical energy consumption, and consideration for analyst safety, as evidenced by the predominance of green color in the pictograms.

## Conclusion

This work introduces a ground breaking selective sensor based on a copolymer of L-dopa and 1,4-phenylenediamine, inspired by mussels, for the voltammetric determination of Bupropion (BUP). This novel sensor can detect BUP in complex matrices such as plasma and in the presence of its co-formulated drug, dextromethorphan, in dosage forms. The study highlights the innovative use of L-dopa and 1,4-phenylenediamine to create a selective sensor for BUP detection. L-dopa, with its carboxylic and phenolic functional groups, provides multiple interaction points with the template molecule, offering significant benefits as a functional monomer. The fabricated electrode exhibited a high degree of selectivity due to the polymer’s strong affinity for BUP, showing a linear response across two concentration ranges of (1.0 × 10^−13^ – 1.0 × 10^−11^ M) with LOD of 3.18 × 10⁻^14^ M. The sensor performed exceptionally well in detecting BUP in the presence of dextromethorphan in dosage forms and spiked human plasma samples. L-dopa and 1,4-phenylenediamine outperformed other well-known monomers like o-phenylenediamine and 4-aminophenol by forming a stable complex with BUP at a 1:1 ratio at pH 7, as confirmed by UV spectrum analysis. The fabricated electrode was thoroughly characterized using cyclic voltammetry and scanning electron microscopy. To enhance the selectivity for the target molecule, various experimental parameters, including the number of cycles, polymerization and binding pH, and scan rate, were optimized. The effective copolymerization provided excellent selectivity for detecting BUP in spiked human plasma. The sensor demonstrated high sensitivity through indirect detection with a ferrocyanide/ferricyanide redox probe and maintained remarkable selectivity for MIP even with higher concentrations of interferents than the analyte. With commendable recoveries and precision, the MIP sensor offers a promising platform for measuring Bupropion in the presence of dextromethorphan in pharmaceutical formulations and spiked human plasma samples.

## Data Availability

The datasets used and/or analysed during the current study available from the corresponding author on reasonable request.
